# Toward an increased reliability of chemical bonding assignment in insulating samples by x-ray photoelectron spectroscopy

**DOI:** 10.1126/sciadv.adi3192

**Published:** 2023-09-15

**Authors:** Grzegorz Greczynski, Oleksandr Pshyk, Lars Hultman

**Affiliations:** Thin Film Physics Division, Department of Physics, Chemistry, and Biology (IFM), Linköping University, SE-581 83 Linköping, Sweden.

## Abstract

X-ray photoelectron spectroscopy (XPS) spectra from solid samples are conventionally referenced to the spectrometer Fermi level (FL). While, in the case of metallic samples, alignment of the sample and the spectrometer FLs can be directly verified from the measured Fermi edge position, thus allowing to assess the surface electrical potential, this is not a workable option for insulators. When applied, it generates a large spread in reported binding energy values that often exceed involved chemical shifts. By depositing insulating amorphous alumina thin films on a variety of conducting substrates with different work functions, we show not only that FL referencing fails but also that the Al_2_O_3_ energy levels align instead to the vacuum level, as postulated in the early days of XPS. Based on these model experiments that can be repeated for all sorts of thin-film insulators, a solution to the binding energy reference problem is proposed for reliable assessment of chemical bonding.

## Teaser

An approach is suggested to increase the reliability of chemical bonding assignment by x-ray photoelectron spectroscopy.

## INTRODUCTION

The increasing importance of surface characterization in essentially all application areas has made x-ray photoelectron spectroscopy (XPS) become one of the most used techniques in modern materials science (*1*). The possibility to analyze chemical bonding and elemental composition for a broad scope of sample types (not only solids of various forms but also gases and liquids) together with substantial instrument improvements (*2*, *3*) over the past half century is a key factor behind this development.

It was realized as early as in the 1970s that XPS spectra recorded from conducting samples are correctly referenced to a common Fermi level (FL), which is established as a result of charge transfer between the sample and the spectrometer. The clear advantage of this approach is that the energy levels from the sample appear at the same binding energy (BE) values irrespective of the sample work function ϕ_SA_, (*4*) making them independent of many difficult-to-control factors such as surface cleanliness (*5*, *6*).

For wide bandgap insulators, however, the charge transfer across the sample/spectrometer interface is very limited even under conditions of x-ray–induced conductivity (*7*); hence, in principle, the insulating sample can be considered to make no electrical contact with the spectrometer (*8*). Under such a circumstance, both sample and the spectrometer share the common vacuum level (VL), which from the theoretical point of view becomes the proper reference level, (*7*, *9*–*12*) allowing for direct comparison of obtained results to gas phase work (*13*) or calculations (*8*). In practice, however, this type of referencing is hindered by surface charging: Electrons leaving the surface due to photoemission are not replaced at a sufficiently high rate owing to poor sample conductivity, which results in an uncontrolled peak shift to higher BE (peak shift from the original position is then a measure of the local surface potential) (*14*–*17*). While the use of low-energy electron guns (flood guns or less-correctly charge neutralizers) (*18*) to counteract charge loss is a very efficient way to acquire spectra even from insulating samples, it does not guarantee that the surface is electrically neutral (*19*).

Another complication is that, under the conditions of VL alignment, the kinetic energy of emitted electrons depends on the sample work function (*9*). Hence, to use the VL referencing, ϕ_SA_ has to be assessed on a case-to-case basis, strictly at the same time XPS spectra are recorded, to ensure that the same surface is analyzed. Such in situ work function measurements are typically done by measuring the secondary electron cutoff corresponding to those electrons that, right before leaving the surface, have the lowest possible energy to overcome the potential barrier and escape into a vacuum (*20*). This is done either by using x-rays or (more commonly) low-energy light sources such as a He lamp (due to the superior line width and higher photon flux) (*6*). Samples are biased negatively to ensure that such low kinetic energy electrons find their way into the analyzer (*20*). However, insulators cannot accommodate the applied negative bias, which, in combination with the surface charging, makes assessment of the secondary electron cutoff essentially impossible (*20*).

To overcome the above problems, the so-called “biased referencing” involving flood guns and internal references (typically in the form of an evaporated dot of a well-characterized metal such as Au or Cu) has been tested (*7*, *21*). Such efforts aimed at developing reliable procedures for measuring the relative change in ϕ_SA_ (e.g., against the well-known standard) and, hence, enable spectra referencing even in the case of insulating samples. However, issues such as band bending at the insulator/metal interface (*22*) and/or the cluster size–induced peak shifts (*23*) hinder the wider implementation of this technique. As a consequence of that, the VL referencing of XPS spectra from solids is rarely seen in modern literature, except for the studies of adsorbates on metal surfaces with sub-monolayer or monolayer coverage (*24*–*26*).

Instead, the vast majority of papers use the condition that surfaces of all air-exposed samples are covered with thin contamination layers composed of carbon, hydrogen, and oxygen (adventitious carbon, AdC). The C 1s spectrum of this layer is recorded and the C-C/C-H component is set at an arbitrarily chosen BE value from the range of 284.0 to 285.6 eV (*27*) and used as an internal reference (*28*, *29*). The latter means that all spectra, including those from the sample itself, are shifted accordingly. This method, however, has generated a long list of complaints (*30*–*34*). Critical reports date back to 1970 but have been widely neglected so the AdC-C 1s method continues to be used. As the topic has been recently reviewed (*27*), we focus here on the most essential problem, which is a trivial observation that the AdC layer is not an inherent part of the sample and, while using the method, a (silent) assumption is made that the sample and the AdC layer share a common FL. For that to be true, charge transfer across the AdC/sample interface is necessary, which with both the sample and the AdC being wide bandgap insulators is very unlikely to take place.

Even in the case of AdC films accumulating on metallic samples, such FL alignment does not happen (*35*, *36*). Instead, energy levels of the AdC layer (including, of course, the C 1s peak used for referencing) align with the VL (*37*), which implies that the position of the C 1s peak depends on the sample work function. As the latter can vary from sample to sample by 2 to 3 eV, any peaks originating from the AdC layer will shift by the corresponding amount, making them useless as an internal reference. This has been shown to hold true for thin-film samples of metals, nitrides, and carbides, all with well-defined Fermi edges, which serve as independent internal references (*38*), thus allowing for direct assessment of the reliability of C 1s referencing. Examples were provided showing that the C 1s method leads to unphysical results (*35*, *39*).

In this contribution, we report on the direct observation of VL alignment in thin films of alumina deposited on electrically conducting substrates with work functions varying in a wide range. The composition and conformality of Al_2_O_3_ films are confirmed by a combination of transmission electron microscopy (TEM) and XPS. The potential influence from other factors such as charging or secondary electrons from the substrate is evaluated by using substrates with different secondary electron yields (Al and W). We conclude that the Al 2p peak positions from the Al_2_O_3_ films are steered by the sample work function, such that their BEs are constant if referenced to the VL. Moreover, the BE of the C 1s peak from AdC that accumulates on top of alumina correlates to the sample work function. Thus, VL alignment appears to be a general condition at both AdC/Al_2_O_3_ and the Al_2_O_3_/substrate interfaces, confirming postulates made in the 1970s and 1980s (*7*, *9*). Based on that, a solution to the charge referencing dilemma is proposed.

## RESULTS

### Characterization of alumina films

**Fig. 1.**
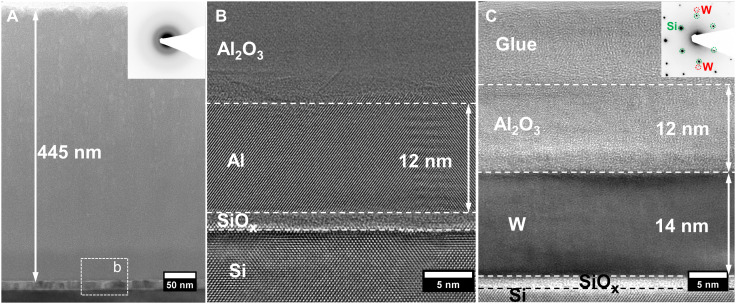
Bright-field cross-sectional TEM images from Al_2_O_3_ samples. (**A** and **B**) Al_2_O_3_/Al/Si and (**C**) Al_2_O_3_/W/Si samples. Corresponding selected area electron diffraction patterns are shown as insets. The Si(001) substrates have a native oxide layer.

Figure 1 shows TEM images together with corresponding selected area electron diffraction (SAED) patterns acquired from Al_2_O_3_/Al/Si and Al_2_O_3_/W/Si samples with different thicknesses of the Al_2_O_3_ layers (see Materials and Methods for details on the film growth). Bright-field TEM (BF-TEM) reveals that dense, featureless, homogenous, and continuous Al_2_O_3_ films are obtained on both Al/Si (Fig. 1, A and B) and W/Si (Fig. 1C) substrates. The thickness of the alumina layers on Al/Si and W/Si substrates is 445 and 12 nm, respectively. SAED pattern from the Al_2_O_3_ film grown on Al/Si substrate (cf. inset in Fig. 1A) shows a distinct diffusive halo ring pattern revealing that the Al_2_O_3_ layer is amorphous. The same conclusion is valid also for the alumina film grown on W/Si (see inset in Fig. 1C), in which case the only reflections seen are those from the Si substrate and the W interlayer. Thus, TEM/SAED investigations confirm conformal growth of amorphous Al_2_O_3_ films irrespective of substrate type and in a wide thickness range. Film growth rates estimated from both TEM images are reasonably close at 1.60 and 1.65 nm/min for alumina layers grown on the Al/Si and the W/Si substrate, respectively.

**Fig. 2.**
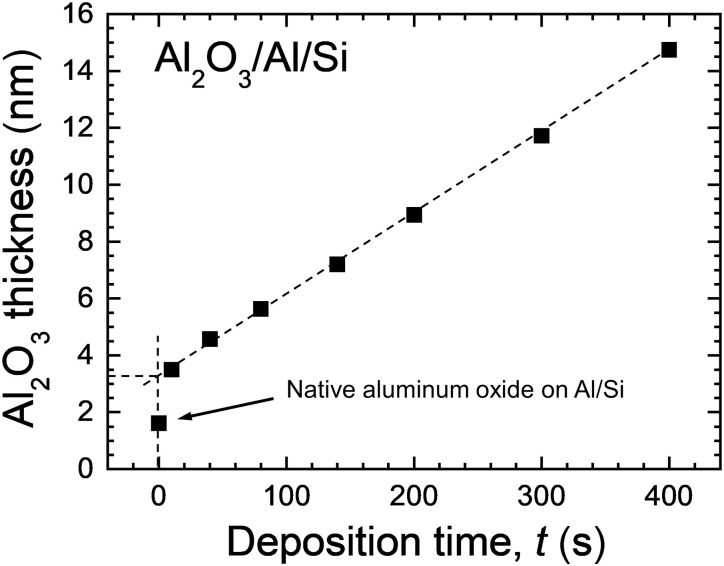
Evidence for the conformal growth of Al_2_O_3_ layers. Thickness of the alumina layer in Al_2_O_3_/Al/Si samples estimated from the relative signal intensities of the Al metal and Al oxide peaks plotted as a function of the deposition time.

Figure 2 shows the thicknesses of alumina layer *d*_Al_2_O_3__ estimated from the relative XPS signal intensities of the Al metal and Al oxide peaks in Al_2_O_3_/Al/Si samples using the formula of Strohmeier (*40*) and the inelastic mean free path of 2.8 nm for Al 2p core-level electrons excited with Al Ka x-rays (*41*), plotted as a function of deposition time. Results are shown only for the thinnest layers, in which case the metallic Al 2p signal from the substrate could still be detected. The analysis is made under the assumption that the attenuation of the x-rays in the alumina layers can be neglected and that the x-ray reflection and refraction are negligibly small (*42*). Clearly, the plot is linear, and the growth rate estimated from the slope is 1.65 nm/min, which is in excellent agreement with values obtained from TEM images. This result confirms that the Al_2_O_3_ layers grow conformally on the macroscopic scale (XPS probes an area of 300 × 700 μm^2^ here) as opposed to island formation (*43*).

The extrapolated oxide thickness for a deposition time of zero seconds gives $d_{{\mathrm{Al}}_{2}O_{3}}^{0}$ = 3.2 nm. This thickness is larger than 1.8 nm, which is the thickness of the native aluminum oxide measured for the reference Al/Si sample exposed to air (after Al film growth) for the same period of time as all other samples. The reason for $d_{{\mathrm{Al}}_{2}O_{3}}^{0}$ being different from zero is that part of the freshly deposited Al film is converted into alumina as a result of the ion-induced intermixing taking place in the surface region once the Al/Si sample is exposed to ~60-eV Ar^+^/O^2+^ ion irradiation.

**Fig. 3.**
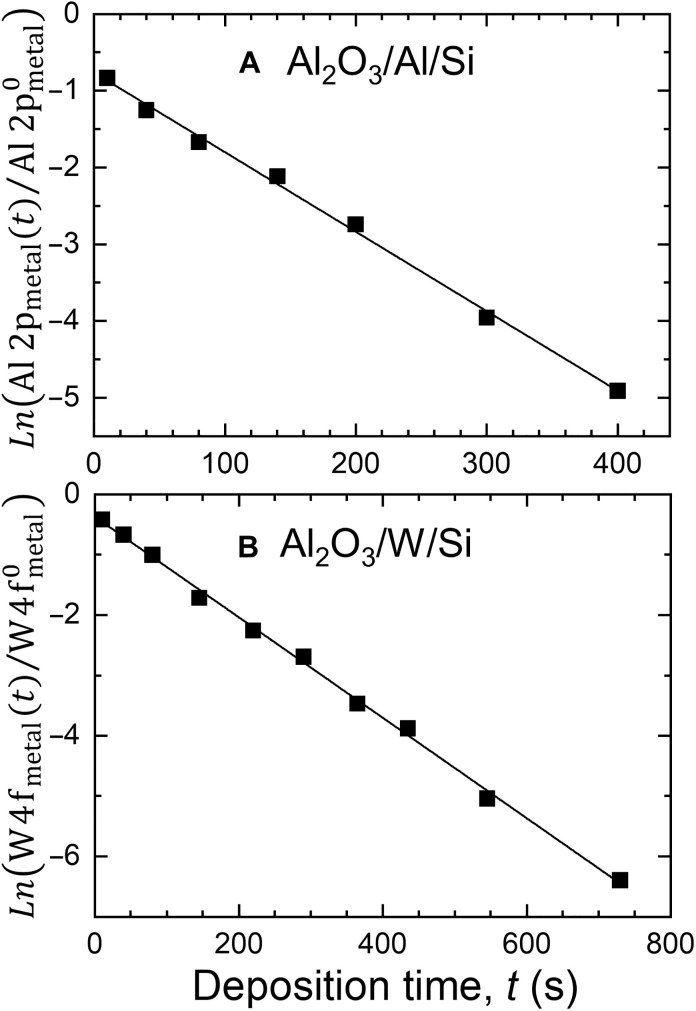
Additional evidence for the conformal growth of Al_2_O_3_ layers. Plots of $Ln\left(\frac{I_{f}(t)}{I_{f}^{0}}\right)$ as a function of deposition time *t*. *I_f_*(*t*) is the XPS intensity of the substrate peak (e.g., metal Al 2p peak in the case of the Al_2_O_3_/Al/Si sample or metal W 4f doublet for Al_2_O_3_/W/Si) corresponding to the sample with Al_2_O_3_ deposition time *t*, and $I_{f}^{0}$ is the intensity of the same substrate peak with the top oxide layer removed by Ar^+^ sputter etch.

Another way to verify the Al_2_O_3_ layer conformity is to plot $Ln\left(\frac{I_{f}(t)}{I_{f}^{0}}\right)$ as a function of deposition time *t*, in which *I_f_*(*t*) is the intensity of the substrate peak (e.g., metal Al 2p peak in the case of the Al_2_O_3_/Al/Si sample or metal W 4f doublet for Al_2_O_3_/W/Si) corresponding to the sample with Al_2_O_3_ deposition time *t*, and $I_{f}^{0}$ is the intensity of the same substrate peak with the top oxide layer removed (by Ar^+^ sputter etch) (*44*). These plots for both the Al_2_O_3_/Al/Si and Al_2_O_3_/W/Si sample series are shown in Fig. 3 (A and B, respectively). The linearity of both plots indicates that the oxide films are conformal. The W 4f signal from the bottom metallic layer of the Al_2_O_3_/W/Si sample is detected for *d*_Al_2_O_3__ ≤ 20 nm (730-s deposition time), while the Al 2p metal peak of the Al_2_O_3_/Al/Si sample is visible for *d*_Al_2_O_3__ ≤ 14.7 nm (400-s deposition time). As electrons originating from both W 4f and Al 2p have similar kinetic energy, this difference cannot be attributed to different mean free paths. Instead, it is explained by ca. 18 times higher photoionization cross section for the excitation of W 4f versus that of Al 2p (*45*), resulting in a substantially higher detection limit in the former case.

The stoichiometry of the Al_2_O_3_ films is assessed from XPS quantitative analyses based on peak areas and instrument-specific sensitivity factors. Samples are analyzed in the as-received state to avoid compositional changes induced by Ar^+^ sputter etching (*46*). In addition to the Al 2p and O 1s signals, the C 1s intensities are included in the analysis to estimate the concentrations of CO and CO_2_ species (parts of AdC) that also contribute to the O 1s spectrum, thus affecting the extracted O/Al ratios. The O/Al ratios after subtracting the AdC contributions are plotted in fig. S1 (see the Supplementary Materials) for the Al_2_O_3_/Al/Si and Al_2_O_3_/W/Si samples series as a function of the Al_2_O_3_ thickness. In the case of thinner oxide films on Al/Si substrates, the metal Al 2p part of the spectra was excluded from the quantifications. We found that O/Al = 1.52 ± 0.03 for Al_2_O_3_/W/Si films with *d*_Al_2_O_3__ ≥ 10 nm, a result which is in excellent agreement with the theoretical value of 1.5. For the thinnest films on W/Si substrates, the O/Al ratio increases above 1.5 due to the contribution of W oxide to the O 1s spectra. In the case of Al_2_O_3_/Al/Si films, O/Al = 1.54 ± 0.06, which also supports the stoichiometric composition of the alumina films.

### Energy-level alignment at the Al_2_O_3_/substrate interface

To investigate the energy-level alignment at the Al_2_O_3_/substrate interface, we analyze the relation between the Al 2p oxide peak position $E_{B}^{F}$ and the sample work function ϕ_SA_ determined by ultraviolet photoelectron spectroscopy (UPS) in the same instrument, directly after XPS analyses (to ensure that the analyzed surface is in the same state). $E_{B}^{F}$ independent of ϕ_SA_ indicates the FL alignment, while the linear relation between the two with a slope equal to one reveals the VL alignment (Schottky-Mott limit) (*47*). Intermediate cases are also possible. This methodology is commonly used to probe the interface electronic structure for organic layers deposited on metallic substrates (*48*).

**Fig. 4.**
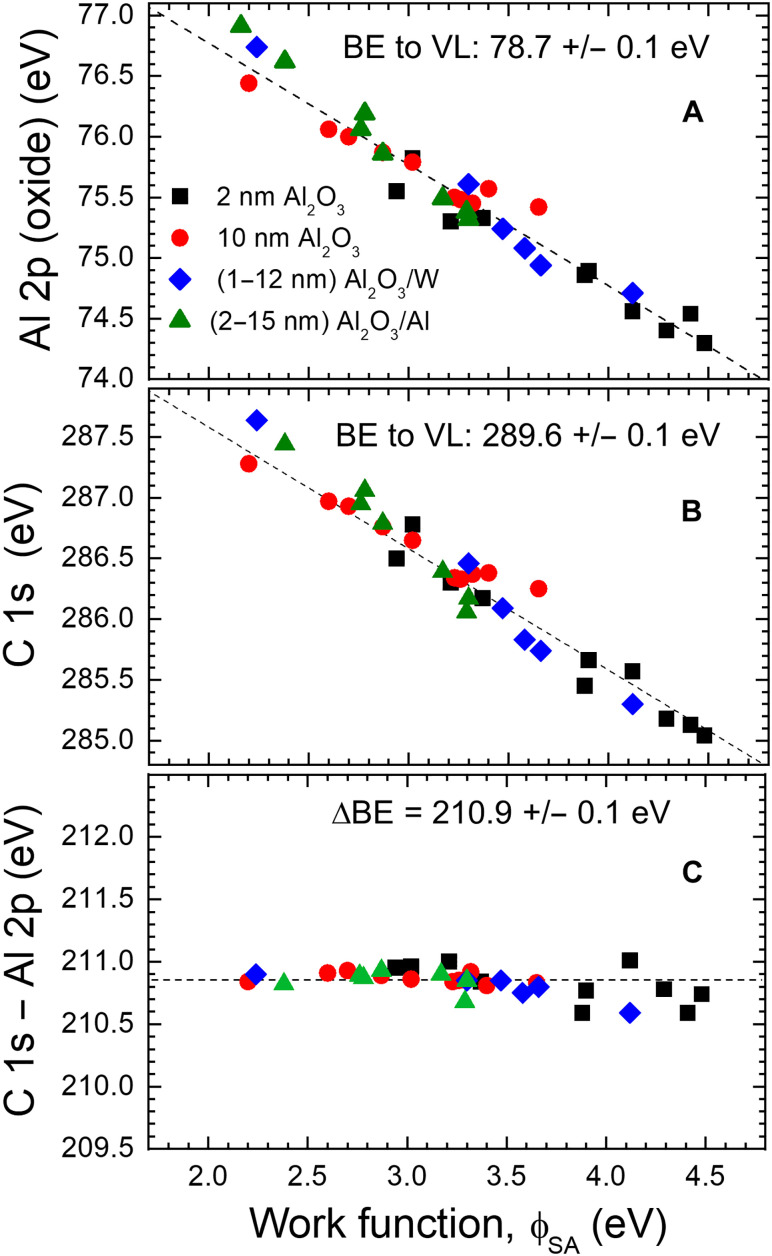
Relation between core-level peak shifts and sample work function. Binding energy of the (**A**) Al 2p oxide peak from Al_2_O_3_, (**B**) C 1s peak from adventitious carbon (the C-C/C-H component) accumulating on top of Al_2_O_3_, and (**C**) the relative shift between both peaks, plotted versus sample work function, obtained from the Al_2_O_3_/X/Si samples (X = Al, W, Si, V, Zr, MoN, HfN, TiN, WN, and VN). Results are shown for ~2- and ~10-nm-thick Al_2_O_3_ layers (i.e., below and above the charging threshold; see the “The role of surface charging” section in Results). In the case of Al and W underlayers, results for several samples are included with 2 ≤ *d*_Al_2_O_3__ ≤ 15 nm.

In Fig. 4A, the BE of the Al 2p oxide peak $E_{B}^{F}$ is plotted versus the sample work function ϕ_SA_ for samples of the type Al_2_O_3_/X/Si with X = Al, W, Si, V, Zr, MoN, HfN, TiN, WN, and VN. For the specimens with ~2-nm Al_2_O_3_ (black squares), ϕ_SA_ varies from 2.9 eV for Al to 4.5 eV for VN. In addition to the ~2-nm Al_2_O_3_ films, results for samples with thicker ~10-nm Al_2_O_3_ layers are also included (red circles) spanning the sample work functions ranging from 2.2 eV (HfN) to 3.7 eV (MoN). To observe possible dependence on the oxide thickness, for two sample types, Al_2_O_3_/Al/Si and Al_2_O_3_/W/Si, data points are shown for 2 ≤ *d*_Al_2_O_3__ ≤ 15 nm. In the former case (green triangles), ϕ_SA_ varies from 2.2 to 3.3 eV, while for the latter series (blue diamonds) 2.2 ≤ ϕ_SA_ ≤ 4.1 eV.

Markedly, the BE of the Al 2p oxide peak from ~2-nm Al_2_O_3_ layers varies by 1.5 eV depending on the underlayer material (from 74.3 eV for VN to 75.8 eV for HfN). Such a variation contradicts the classical picture in which a given chemical state is associated with a specific BE value (*4*). Moreover, the magnitude of the Al 2p shifts shown in Fig. 4A is comparable to that of related chemical shifts (e.g., the BE difference between the Al 2p peak of Al_2_O_3_ and AlN is 1.4 eV) (*49*), making the essential part of XPS analysis, i.e., bonding assignment, very challenging if not impossible.

The solution to the above dilemma is based on the observation that a linear trend exists between $E_{B}^{F}$ and ϕ_SA_ such that the sum $E_{B}^{F}$ + ϕ_SA_ is constant at 78.7 ± 0.1 eV. This result means that the energy levels of Al_2_O_3_ align to the VL, for the alumina thickness range tested (2 ≤ *d*_Al_2_O_3__ ≤ 12 nm; cf. also the “Consequences of the VL alignment for spectra referencing” section in Results). Thus, in this respect, they behave the same way as AdC films that are found on all surfaces exposed to air (*35*, *37*). The latter point is also verified for the current set of samples. Figure 4B shows the plot of C 1s BE (the C-C/C-H component) versus ϕ_SA_ confirming a linear relationship with the C 1s BE referenced to VL of 289.6 ± 0.1 eV, in perfect agreement to 289.58 ± 0.15 eV previously reported for nearly 100 thin-film samples (*37*). The BE of the Al 2p oxide peak relative to the C 1s peak of AdC is then ~210.9 ± 0.1 eV (cf. Fig. 4C) and remains independent of the sample work function and alumina layer thickness for *d*_Al_2_O_3__ ≤ 12 nm. Both AdC and Al_2_O_3_ align to VL as, in both cases, there are not enough free charges to establish the FL alignment to the spectrometer. This implies that, in the case of thin oxide layers, VL is the correct referencing level as was postulated already back in the early days of XPS (*7*, *9*) but, to the best of our knowledge, never explicitly demonstrated experimentally. Further implications for the charge referencing of insulating samples are discussed in the “Consequences of the VL alignment for spectra referencing” section in Results.

### The role of surface charging

Peak shifts from insulating layers such as Al_2_O_3_ are commonly observed in XPS due to surface charging. Thus, we evaluate the potential influence of this effect on the Al 2p and C 1s peak shifts shown in Fig. 4.

It has been reported already in 1980 by Lewis and Kelly (*9*) that compensating charge from the flood gun distorts the oxide part of the Al 2p spectra from Al_2_O_3_ provided that samples are grounded, while the Al 2p metal peak remains unaffected. This effect, commonly observed not only for alumina but also for other types of thin oxides and metallic substrates, (*19*, *50*, *51*) occurs since the extra charge cannot drain away through the insulating oxide layer and resides at the surface making it negative. As a result, the oxide peak moves toward the (unaffected) metal peak. This observation, which is confirmed by different laboratories using several common instruments, appears to be a determining test for the presence (or the lack) of surface charging.

**Fig. 5.**
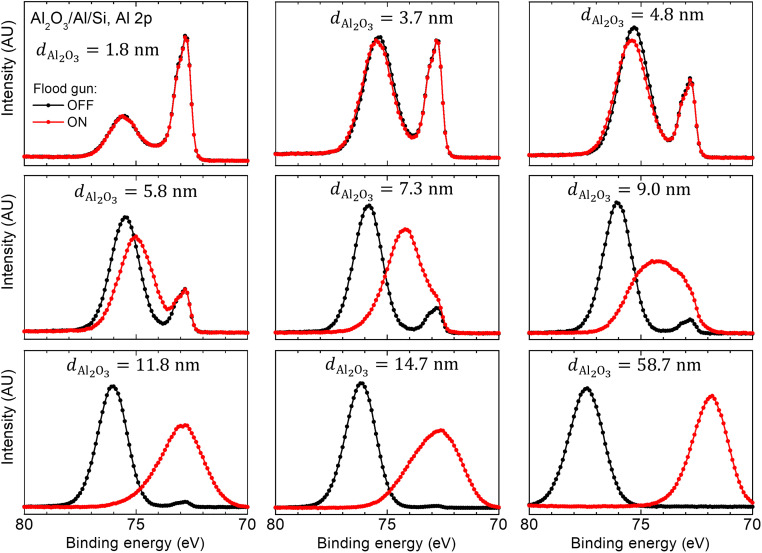
Investigating the role of surface charging. Al 2p spectra from Al_2_O_3_/Al/Si sample series with alumina layer thickness *d*_Al_2_O_3__varying from 1.8 to 58.7 nm. Data are acquired with (red) and without (black) flood gun. AU, arbitrary units.

Al 2p spectra recorded with and without charge compensation for samples from the Al_2_O_3_/Al/Si series with the alumina thickness *d*_Al_2_O_3__ varying from 1.8 to 58.7 nm are shown in Fig. 5. Corresponding changes in the full width at half maximum of the Al 2p oxide peak are included in the Supplementary Materials (fig. S2). Clearly, for *d*_Al_2_O_3 __≲ 5 nm, no substantial spectral modification is detected, which indicates that the oxide is thin enough to allow for the surplus charge from the flood gun to drain away to the substrate such that the entire structure is maintained at the same electrical potential. Noticeable change between spectra recorded with and without the flood gun is observed for the sample with *d*_Al_2_O_3__ = 5.8 nm, which marks the onset of charging. The spectral evolution is then typical (*9*, *19*, *50*, *52*): The signal from the oxide broadens (cf. fig. S2) and moves to lower BE and eventually overlaps with the Al 2p peak from the substrate, which is observed at a fixed BE irrespective of *d*_Al_2_O_3__.

**Fig. 6.**
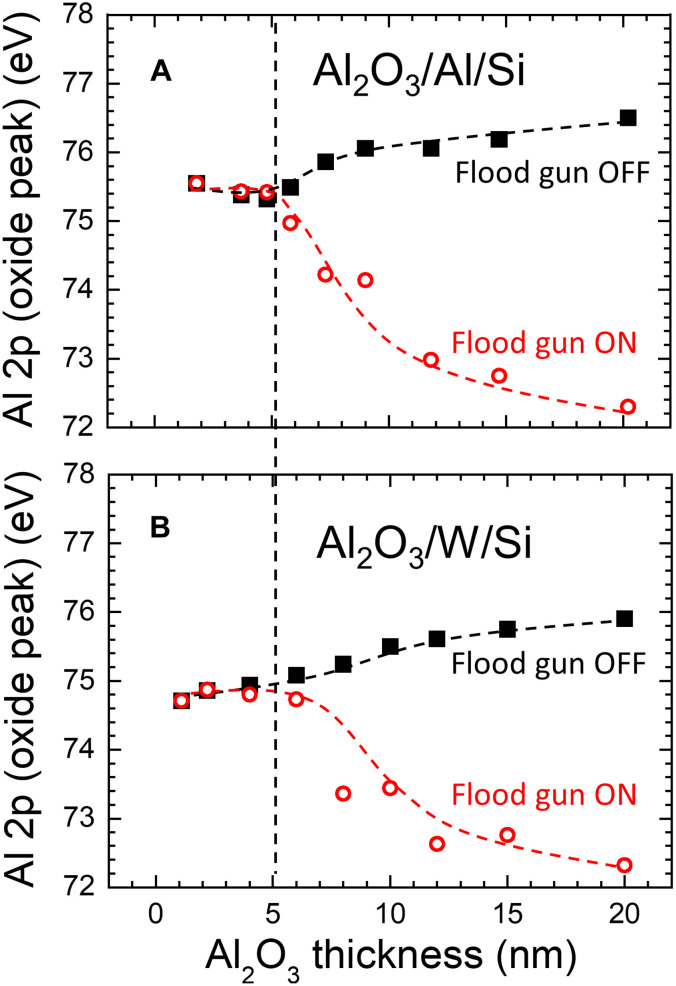
Determination of the critical Al_2_O_3_ thickness. Binding energy of the Al 2p oxide peak as a function of the Al_2_O_3_ thickness for (**A**) Al_2_O_3_/Al/Si and (**B**) Al_2_O_3_/W/Si samples. In both cases, measurements are performed with and without flood gun to determine the onset of surface charging (indicated with vertical dashed lines).

BE shifts of the Al 2p oxide peak as a function of the oxide thickness are summarized in Fig. 6 (A and B) for two sample series: Al_2_O_3_/Al/Si and Al_2_O_3_/W/Si, respectively. In both cases, measurements are performed with and without the low-energy electron gun (sometimes also called a flood gun). Considering the Al_2_O_3_/Al case, the position of the oxide peak is constant at 75.5 ± 0.1 eV up to *d*_Al_2_O_3__ ≈ 5 nm, irrespective of whether the flood gun is used or not. The alumina is so thin within this range that the positive charge resulting from photoemission is completely neutralized by the secondary substrate electrons with mean free paths on the order of 2.8 nm (Al 2p core-level electrons excited with Al Ka x-rays) (*41*). The Al_2_O_3_ layer is also thin enough to allow for the surplus negative charge deposited on the surface by the flood gun to drain away to the substrate such that the measured position of the Al 2p oxide peak and its shape is the same no matter if the flood gun is used or not.

If the oxide thickness exceeds 5 nm, the position of the Al 2p oxide peak becomes dependent on whether the flood gun is used or not (cf. Fig. 6), thus marking the critical oxide thickness for which the presence of surface charging in the Al oxide layer can be undoubtedly confirmed. With no charge compensation, the oxide peak shifts to higher BE (75.9 eV with *d*_Al_2_O_3__ = 7 nm), while, with the flood gun on, the shift is toward lower BE (first to 75.0 eV with *d*_Al_2_O_3__ = 6 nm and then to 74.2 eV with *d*_Al_2_O_3__ = 7 nm). In the former case, the oxide layer becomes positively charged, which is ascribed to the decreasing flux of the substrate electrons (as there is no obvious reason why the fluxes of compensating electrons coming from other sources should be affected by a small change in the oxide thickness), that decay exponentially with increasing the oxide thickness ~exp(−*d*_Al_2_O_3__/λ) (for such thin oxide layers, the absorption of x-rays in the oxide can be neglected). In the case with the flood gun on, the Al 2p oxide peak shifts to lower BE due to the fact that the surface becomes negatively charged (or overcompensated) as the electron flux delivered by the flood gun cannot freely drain away to the substrate through (now) too thick an oxide layer. The magnitude of both effects described above increases with increasing *d*_Al_2_O_3__, which leads to the increasing splitting between the BE of the oxide peak measured with and without charge compensation (cf. Fig. 6A).

The BE values for the Al 2p oxide peak shown in Fig. 6A are in very good agreement with previously published results for the Al_2_O_3_/Al system by Baer *et al.* (*19*). In that paper, oxide layers forming on Al after exposure to NaCl solutions (with pH 10 adjusted with NaOH) for different times (5, 15, and 30 min) were analyzed. An Al oxide peak was found at 75.7, 76.5, and 76.9 eV for *d*_Al_2_O_3__ = 6.4, 22.0, and 36.0 nm, respectively (no charge compensation used). Linear extrapolation of our data gives 75.65 eV for *d*_Al_2_O_3__ = 6.4 nm, 76.54 eV for *d*_Al_2_O_3__ = 22.0 nm, and 76.81 eV for *d*_Al_2_O_3__ = 36.0 nm. Thus, an agreement between the two sets of experiments is better than 0.1 eV, which is noteworthy given the completely different oxide preparation method and the different instrument used by Baer *et al*. (*19*).

Qualitatively similar behavior to the one described for the Al_2_O_3_/Al/Si case is also observed for Al_2_O_3_ layers grown on top of the W/Si substrate (see Fig. 6B). The critical alumina thickness that marks the onset of surface charging is the same as for the Al_2_O_3_/Al/Si case, i.e., *d*_Al_2_O_3__ ≈ 5 nm, after which the peak shifts to either higher or lower BE, depending on whether the flood gun is off or on. Thus, it appears from the results in Fig. 6 that the onset of photoemission-induced charging is independent of the substrate type and, in particular, is not affected by the intensity of the secondary electron flux from the substrate.

A distinct difference between the two sample series, Al_2_O_3_/Al/Si and Al_2_O_3_/W/Si, is that the Al oxide peak for the thinnest layers appears at 75.5 ± 0.1 eV and 74.8 ± 0.1 eV, respectively. Markedly, the ~0.7-eV difference is independent of whether the flood gun is used or not, making it clear that the reason for this peak shift is not related to charging effects. Instead, it is easily explained by the difference between Al_2_O_3_/Al/Si and Al_2_O_3_/W/Si work functions, which for charging-free films (*d*_Al_2_O_3__ ≲ 5 nm) are 3.1 ± 0.1 and 3.9 ± 0.1 eV, respectively. Thus, the Al 2p BE is constant with respect to the VL at 78.7 ± 0.1 eV, as described in the “Energy-level alignment at the Al_2_O_3_/substrate interface” section in Results.

### The role of secondary electron fluxes from the substrate

**Fig. 7.**
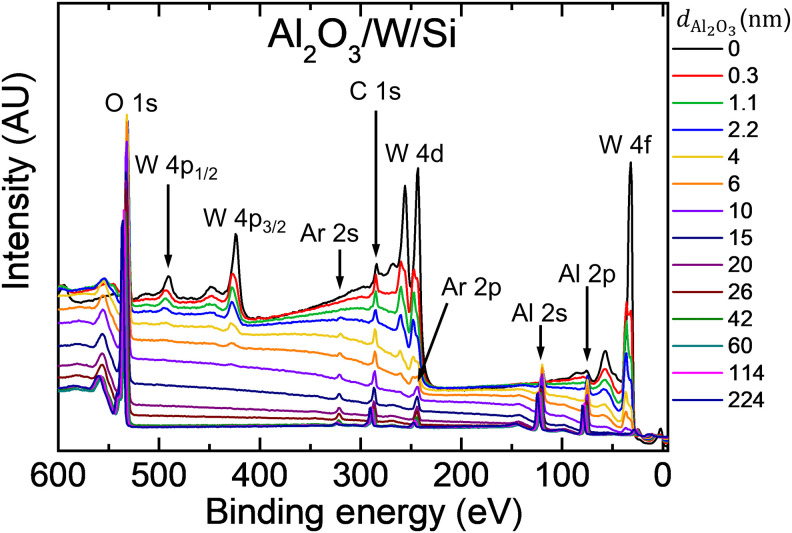
Evidence for the presence of secondary electrons from the substrate. Survey scans recorded from the Al_2_O_3_/W/Si sample series with the alumina thickness varying in the range of 0 ≤ *d*_Al_2_O_3__ ≤ 224 nm.

In this section, we consider the role of secondary electrons from the substrate. Their presence is evident in Fig. 7, showing survey scans recorded from the Al_2_O_3_/W/Si sample series with alumina thicknesses varying in the range of 0 ≤ *d*_Al_2_O_3__ ≤ 224 nm. For the thinner layers, *d*_Al_2_O_3__ ≤ 20 nm, all W peaks show an increased background on the high BE side due to inelastically scattered electrons originating from the W substrate. The effect is best visible for the W 4d and W 4f core levels. The fast drop in the inelastic background intensity with increasing *d*_Al_2_O_3__ is caused by an exponential attenuation of the substrate signal which varies as ∼exp(−*d*_Al_2_O_3__/λ). For *d*_Al_2_O_3__ > 20 nm, the change in the background shape is small, and the inelastic background is completely absent for alumina films thicker than 42 nm (in which case, the attenuation factor for W 4f electrons excited with Al Ka x-rays is 3 × 10^−7^).

**Fig. 8.**
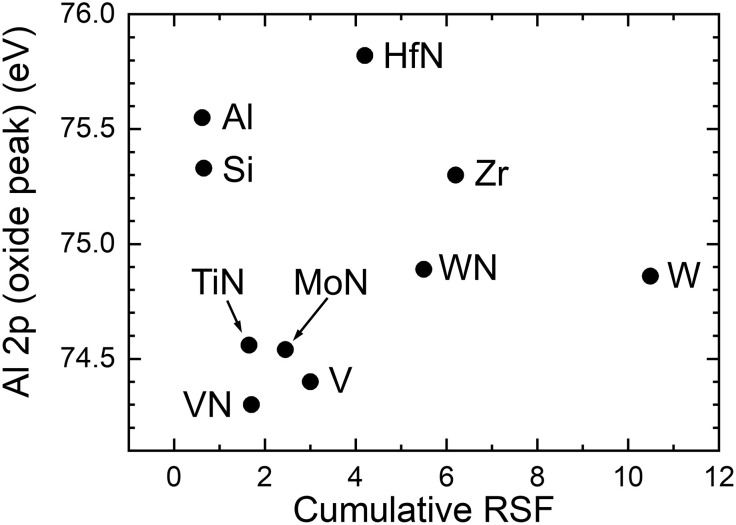
Investigating the role of secondary electron fluxes from the substrate. Binding energy of the Al 2p oxide peak from ~2-nm-thick Al_2_O_3_ layers grown on X/Si (X = Al, W, Si, V, Zr, MoN, HfN, TiN, WN, and VN) plotted as a function of cumulative RSF for core levels of the substrate material.

The secondary electron flux can be quantified for a given substrate type by the cumulative sum Σ_RSF_ of relative sensitivity factors (RSFs) for all core-level electrons excited with Al Ka x-rays. Σ_RSF_ differs substantially between different substrates due to differences in the photoemission yield. For example, Σ_RSF_ is nearly 17 times higher for W than for Al. Thus, one can expect that peaks from the alumina layer shift to lower BE if the Al_2_O_3_ layer is exposed to higher fluxes of substrate electrons, in analogy to shifts induced by low-energy electrons from the flood gun (see Figs. 5 and 6). There are, however, arguments that refute this hypothesis:

1) As demonstrated in “The role of surface charging” section in Results, the flood gun has no effect on the position of Al 2p oxide peaks for *d*_Al_2_O_3__ < 5 nm. Within this thickness range, the BE of the Al oxide peak is constant revealing that the surplus charge deposited on the surface can dissipate to the substrate. If high fluxes of low-energy electrons supplied by the flood gun have no effect on the peak positions in alumina layers thinner than 5 nm, then why should substrate electrons have any impact?

2) The BE offset between the Al oxide peak from Al_2_O_3_/W/Si and Al_2_O_3_/Al/Si samples is observed for alumina films as thick as 60 nm and disappears for *d*_Al_2_O_3__ > 100 nm. Thus, the effect is present even though no inelastically scattered electrons from the substrate are detected (cf. Fig. 7).

3) There is no correlation between the BE of the Al 2p oxide peak and Σ_RSF_. Figure 8 shows results obtained from ~2-nm Al_2_O_3_ films deposited on a variety of substrates including Al, W, Si, V, Zr, MoN, HfN, TiN, WN, and VN. For Al_2_O_3_/TiN/Si and Al_2_O_3_/VN/Si, the oxide peak is at even lower BE than in the case of Al_2_O_3_/W/Si, 74.6 and 74.4 eV, respectively, although the corresponding Σ_RSF_ values are much lower (1.65 and 1.7 versus 10.5 for W). Similarly, the Al oxide peak is at 75.8 eV for Al_2_O_3_/HfN/Si sample with relatively high Σ_RSF_ (4.2), i.e., at even higher BE than that obtained for the Al_2_O_3_/Al/Si case. Thus, Al 2p oxide peak shifts cannot be explained by varying fluxes of substrate electrons.

### Consequences of the VL alignment for spectra referencing

**Fig. 9.**
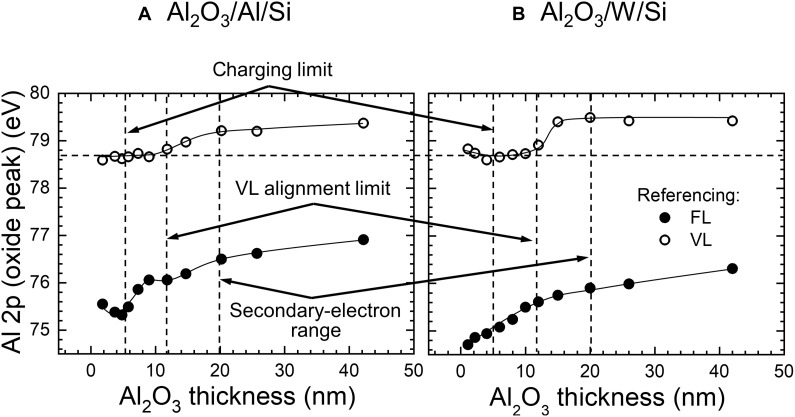
Comparison of vacuum level and Fermi level referencing. Binding energy of the Al 2p oxide peak plotted as a function of the Al_2_O_3_ thickness for (**A**) Al_2_O_3_/Al/Si and (**B**) Al_2_O_3_/W/Si samples using FL and VL referencing.

Clear benefits of VL referencing are illustrated in Fig. 9 (A and B) for the case of the Al_2_O_3_/Al/Si and Al_2_O_3_/W/Si sample series. The BE of the Al 2p oxide peak is plotted as a function of Al_2_O_3_ thickness using both FL (i.e., measured $E_{B}^{F}$ values) and VL (i.e., $E_{B}^{V}=E_{B}^{F}+\phi_{\mathrm{SA}}$) referencing. In the former case, the Al oxide peak position depends both on the substrate type and the oxide thickness *d*_Al_2_O_3__. In contrast, with VL referencing, the Al 2p oxide peak is at 78.7 ± 0.1 eV for 0 ≤ *d*_Al_2_O_3__ ≤ 12 nm, irrespective of the substrate type, which allows us to maintain the basic XPS paradigm that the given chemical state is associated with a specific BE value (and not the whole range). The BE of that peak does not vary up to *d*_Al_2_O_3__ ≈ 12 nm, which means that the VL alignment is preserved well above the charging limit of 5 nm (cf. the “The role of surface charging” section in Results) but is disrupted before the secondary electron range of ~20 nm is reached (see the “The role of secondary-electron fluxes from the substrate” section in Results).

**Fig. 10.**
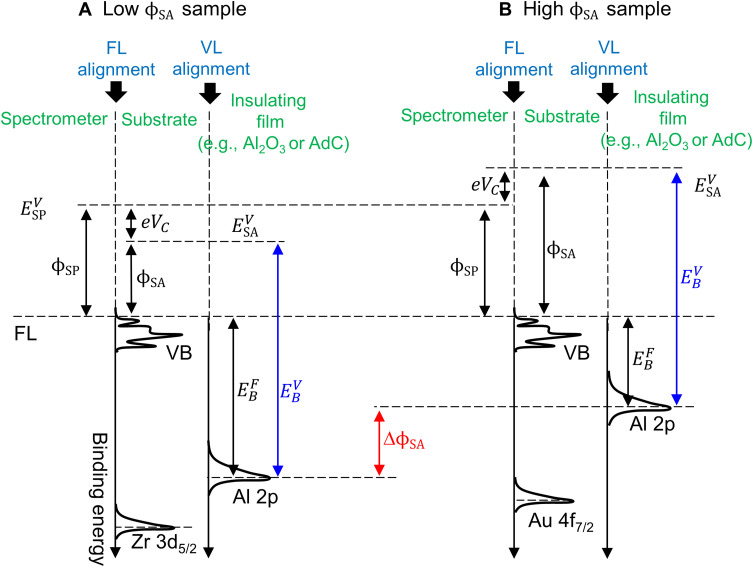
The connection between core-level peak shifts and sample work function for the case of insulating films on conducting substrates. Schematic energy-level diagrams for the case of (**A**) low and (**B**) high work function samples. FL alignment is valid at the substrate/spectrometer interface, while alumina aligns to the VL of the substrate. Because of that sample VL $E_{\mathrm{SA}}^{V}$ shifts with respect to the FL and so does the Al 2p peak from the alumina layer ($E_{B}^{F}$). The change in $E_{B}^{F}$ is determined by the work function difference between the two samples Δϕ_SA_. However, the BE of the Al 2p peak referenced to the sample VL $E_{B}^{V}$ is constant.

The reason for shifts of core-level peaks from insulating layers deposited on conducting substrates with variations in the sample work function is schematically illustrated in Fig. 10 for the case of low and high ϕ_SA_ (Fig. 10, A and B, respectively), assuming no additional complications such as, e.g., charge accumulation at interfaces or dipole layer formation. If good electrical contact is established between the substrate and the spectrometer, then FL alignment takes place at the substrate/spectrometer interface as a result of a negative charge flow from the body with a lower work function to the one with a higher work function. Thus, for the case of the low work function sample (ϕ_SA_ < ϕ_SP_; Fig. 10A), upon contact, electrons flow from the substrate to the spectrometer, resulting in a contact potential difference *V_c_* with the spectrometer side charging negatively until equilibrium is reached and the FL on both sides is aligned. In the high work function case (ϕ_SA_ > ϕ_SP_; Fig. 10B), the charge flows in the opposite direction. Whether the above takes place or not can be experimentally verified by recording the XPS spectrum in the vicinity of the FL and observing the position of the FL cutoff. The latter corresponds to the middle of the rapid drop in the density of states or, more precisely, to the center of the peak obtained after taking the signal derivative (*53*). In the case of a complete FL alignment, the Fermi edge should be at 0 eV on the BE scale.

In contrast to the substrate/spectrometer interface, no charge flow takes place at the interface between the substrate and the insulating film (e.g., nonconducting oxide or AdC layer or both). Hence, both the substrate and the oxide share a common VL $E_{\mathrm{SA}}^{V}$. $E_{\mathrm{SA}}^{V}$ is lower than the spectrometer VL $E_{\mathrm{SP}}^{V}$ for the low work function sample (cf. Fig. 10A), while $E_{\mathrm{SA}}^{V}>E_{\mathrm{SP}}^{V}$ in the case of high ϕ_SA_ (cf. Fig. 10B). As a result, all energy levels from the top insulating film are affected by ϕ_SA_. What this means, in practice, is that for the Al_2_O_3_ layer, the position of the Al 2p oxide peak referenced to the spectrometer FL, $E_{B}^{F}$, is high if the oxide is grown on the low work function substrate and low if the high work function substrate is used. The Al 2p peak shift between the two cases is then equal to the work function difference Δϕ_SA_. As the latter is of the same order as the chemical shifts, the conventional referencing to the FL prevents meaningful analysis of the sample chemistry. That can be resolved in the case of thin-film samples by using VL referencing, as the position of the Al 2p oxide peak referenced to the VL, $E_{B}^{V}$, is constant for *d*_Al_2_O_3__ ≲ 12 nm (cf. Figs. 9 and 10).

The above scenario is valid for thinner films, *d*_Al_2_O_3__ ≲ 12 nm, for which charging effects are not severe. While the critical thickness may vary from one insulator to another, the experiments described here can, in principle, be conducted for any material system that can be obtained in thin-film form (not necessarily by vacuum-based techniques). This approach would allow one to obtain reliable core-level BE values (referenced to the VL) for peaks from such insulators (e.g., 78.7 eV for the Al 2p peak from amorphous Al_2_O_3_; cf. Fig. 9). Such a library could fulfill the purpose of an internal charge reference for thicker samples that need to be analyzed with external charge compensation.

Up to this point, we assumed that information about the sample work function can be obtained. If that, for whatever reason, is not the case, then we propose to drop the absolute energy referencing practiced today for the sake of substantially improved accuracy of the chemical state determination. Since we showed that the energy levels from both the AdC layer and the insulator align to the VL, the BE difference between sample signals and the C 1s peak of AdC (the C-C/C-H component) is constant (e.g., 210.9 eV for the Al 2p peak of Al_2_O_3_; cf. Fig. 4C). Knowing that, one can assess the chemical state from thicker insulating samples which require charge compensation simply by measuring the position of spectral peaks relative to that of the C 1s AdC peak and comparing the obtained results to reference values derived from thin films following the same procedures as described here. Hence, the work function measurement is not necessary. Our measurements conducted on alumina layers of varying thickness indicate that the C 1s – Al 2p BE difference remains constant at 210.9 eV (despite large C 1s and Al 2p absolute shifts due to charging) at least up to *d*_Al_2_O_3__ = 467 nm, which is the thickest film tested (cf. figs. S3 to S5). Such analysis relies on the relative peak shifts and is, thus, conceptually different from the commonly practiced absolute referencing which places the C 1s peak at a specific BE value (*28*, *29*).

While the absolute BE values can be precisely determined for metallic samples, the procedures applied for insulators are questionable. In the latter case, the surface electrical potential, which directly affects peak positions, is not well defined irrespective of whether measurements are performed with or without charge compensation and with samples grounded or isolated from the instrument (*50*). In a typical workflow, one arbitrarily sets the C 1s peak at 284.0 to 285.6 eV [see (*27*)] and compares measured peak positions to databases containing BE values that often show an unacceptably large spread (e.g., from 73.6 to 75.9 eV for the Al 2p peak of Al_2_O_3_) (*54*). It does not come as a surprise then that with such wide energy ranges, often larger than the involved chemical shifts, the risk of spectral misinterpretation is large. In addition, the common referencing procedure completely neglects the fact that AdC aligns with the VL (*35*–*37*), which in the case of a sample with a relatively low work function leads to nonsensical results such as finding the occupied density of states above the FL (*35*, *39*).

Problems of the conventional method are resolved either by referencing to VL (in cases where the sample work function can be assessed experimentally) or (if the measurement of the sample work function is not feasible) by use of the relative referencing method outlined in this work. In the latter case, we postulate abandoning the absolute referencing method for the sake of substantially improved accuracy in chemical state determination, which is the primary reason for doing XPS anyway.

## DISCUSSION

We studied the energy-level alignment of thin Al_2_O_3_ films deposited by magnetron sputtering on a variety of electrically conducting substrates X/Si with X = Al, W, Si, V, Zr, MoN, HfN, TiN, WN, and VN. By comparing Al 2p peak positions and spectral shapes recorded with and without charge compensation, we determined the critical alumina thickness above which surface charging occurs. For Al_2_O_3_ layers thin enough to avoid charging, we then show that the Al 2p BE $E_{B}^{F}$ exhibits a linear relationship with the sample work function ϕ_SA_, assessed from the secondary electron cutoff, such that the sum $E_{B}^{F}+\phi_{\mathrm{SA}}$ is constant at 78.7 ± 0.1 eV. The VL alignment holds up to an Al_2_O_3_ thickness of 12 nm, while the first indications of charging are observed for alumina layers thicker than 5 nm.

On the same account, the BE of the C-C/C-H peak in the C 1s spectra of AdC accumulating on top of Al_2_O_3_ follows changes in ϕ_SA_ such that its position is constant with respect to the VL at 289.6 ± 0.1 eV, in perfect agreement with our earlier results for conducting layers.

Thus, VL alignment is a general condition at both AdC/Al_2_O_3_ and Al_2_O_3_/substrate interfaces. Our results fully confirm assessments made back in the 1970s and 1980s (*9*, *10*, *12*) that the VL is the correct reference level in the absence of charge transfer, as is characteristic of insulators. The commonly applied practice of referencing to the hydrocarbon C 1s peak of AdC, by setting it at the predefined BE value (*28*, *29*), inherently assumes FL alignment and is, thus, incorrect as it results in a substantial uncertainty of the chemical bonding assignment.

The present work offers a possible solution to the BE referencing problem as the experiments described here can be conducted for other types of thin-film insulators with thicknesses below the charging limit. The ultimate goal would be a library of core-level peak positions referenced to (i) the sample VL (for absolute referencing in case the sample work function can be measured) and (ii) the hydrocarbon C 1s peak of AdC (for relative referencing in all cases where work function value cannot be obtained). The latter should not be confused with the unreliable practice of absolute referencing, which relies on setting the C 1s peak at a predefined BE value. For even better control and reproducibility, such analyses might be conducted in situ, i.e., without exposing freshly deposited films to air.

For cases where information about the sample work function can be obtained, VL referencing is advised as it allows a user to maintain the basic concept of XPS, i.e., that a given chemical state is associated with only one BE value. The additional advantage is that such referenced BE values can be directly compared to the gas phase measurements. If, however, a work function measurement is not feasible, we propose to drop the absolute energy referencing practiced today for the sake of substantially improved accuracy in chemical-state determination, which is the primary goal of XPS analyses (*55*). The latter scenario is based on the observation that the BE difference between peaks from an insulator and the C 1s peak of the related AdC layer is constant (confirmed here for alumina films in the thickness range of 2 ≤ *d*_Al_2_O_3__ ≤ 467 nm), as both share a common VL. One can, thus, assess the chemical state from insulating samples by measuring core-level positions relative to that of the C 1s AdC peak and comparing reference values derived from thin films following the procedures described here. The absolute BE of the C 1s peak has no significance as it is controlled by the sample work function.

Irrespective of which method is applied, the reliability of XPS data analysis can be further enhanced by including reference samples (often with simpler composition, thus easier to interpret) (*53*), focusing on relative spectral changes in sample series (*56*), and paying attention to the sample history (e.g., minimize the time between sample preparation and analysis as well as careful sample handling and storage) (*57*, *58*).

The approach proposed in this contribution obviously does not resolve all complications related to XPS analyses of complex materials. In particular, problems arising due to charge accumulation at interfaces, dipole layer formation, or differential charging in inhomogeneous systems need to be addressed on a case-by-case basis.

## MATERIALS AND METHODS

The thin-film samples used in this study are all grown on Si(001) substrates by magnetron sputtering in a multicathode CC800/9 CemeCon AG system. Substrates are first coated with ~50-nm-thick Al or W layers using rectangular 8.8 × 50 cm^2^ elemental targets and a pure Ar atmosphere with a total pressure of 3 mTorr (0.4 Pa). Immediately after that, the gas composition is changed to an Ar/O_2_ mixture with an O_2_-to-Ar flow ratio of 0.3 and with the same total pressure. A target-to-substrate distance of 18 cm is used in all cases. No intentional heating is used so the substrate temperature during growth is determined by the plasma heating and does not exceed 80°C. To minimize the influence of venting temperatures on the thickness of the surface oxide layer (*59*), all specimens are allowed to cool down to room temperature before exposure to the laboratory atmosphere. The underlayers are grown by direct current magnetron sputtering, while high power impulse magnetron sputtering is used for the Al_2_O_3_ growth (the average power, the pulse length, and the pulsing frequency are at 1 kW, 50 μs, and 4000 Hz, respectively). The synchronized substrate bias with an amplitude of −60 V and a pulse length of 200 μs is used. Al_2_O_3_ film thickness is varied from 2 to 467 nm by varying the deposition time.

Additional sets of Al_2_O_3_ films with thicknesses of 2 and 10 nm are also grown on top of Si, V, Zr, MoN, HfN, TiN, WN, and VN underlayers previously deposited on Si(001) substrates in the same film growth sequence (i.e., without breaking the vacuum). Process parameters used to deposit all nitrides are the same as in our previous studies (*60*).

Following the growth, samples are immediately transferred to the load lock chamber of an Axis Ultra DLD instrument from Kratos Analytical (UK) such that the air exposure time is shorter than 2 min. The base pressure during XPS analyses is better than 1.1 × 10^−9^ Torr (1.5 × 10^−7^ Pa). Monochromatic Al Kα radiation (hν = 1486.6 eV) is used with the anode power set to 150 W and all spectra are recorded at normal emission angle. The analyzer pass energy is 20 eV, which yields a full width at half maximum of 0.55 eV for the Ag 3d_5/2_ peak. The area analyzed by XPS is 0.3 × 0.7 mm^2^. Spectrometer calibration is verified by measuring Au 4f_7/2_, Ag 3d_5/2_, and Cu 2p_3/2_ peak positions from sputter-etched Au, Ag, and Cu samples and comparison to the recommended ISO standards for monochromatic Al Ka sources (*61*).

No charge referencing is performed as the intention is to study the peak shifts induced by surface charging. It is observed though that, for samples with thinner Al_2_O_3_ films, the substrate signals appear at correct positions [e.g., the metal Al 2p peak at 72.9 eV in the case of Al_2_O_3_/Al/Si(001) specimens] (*62*), confirming the FL alignment at the substrate/spectrometer interface. A low-energy electron gun is used in some experiments as described in the “The role of surface charging” section in Results.

UPS measurements are conducted with unmonochromatized He I radiation (hν = 21.22 eV) in the same instrument as used for XPS analyses. A −10-V bias is applied to the sample stage. The sample work function is estimated from the secondary electron cutoff energy in the He I UPS spectra by a linear extrapolation of the low kinetic energy electron tail toward the BE axis (*63*, *64*). The measurement on the sputter-etched reference Au sample gives 5.30 eV, confirming the correct calibration (*63*).

An FEI Tecnai G2 TF 20 UT FEG microscope is used to obtain bright-field TEM images and SAED patterns. TEM specimens are prepared by mechanical polishing followed by ion milling in a Gatan precision ion miller.
