# Supplementary material for: Toward an increased reliability of chemical bonding assignment in insulating samples by x-ray photoelectron spectroscopy
Source: Sci Adv. 2023 Sep 15;9(37):eadi3192. doi: 10.1126/sciadv.adi3192 (PMC10881046; doi:10.1126/sciadv.adi3192)
Supplement: Supplementary file 1 — Figs. S1 to S5 [file sciadv.adi3192_sm.pdf]

Science Advances
AAAS

Supplementary Materials for

Toward an increased reliability of chemical bonding assignment in insulating samples by x-ray photoelectron spectroscopy

Grzegorz Greczynski *et al.*

Corresponding author: Grzegorz Greczynski, grzegorz.greczynski@liu.se

Sci. Adv. **9**, eadi3192 (2023)
DOI: 10.1126/sciadv.adi3192

This PDF file includes:

Figs. S1 to S5

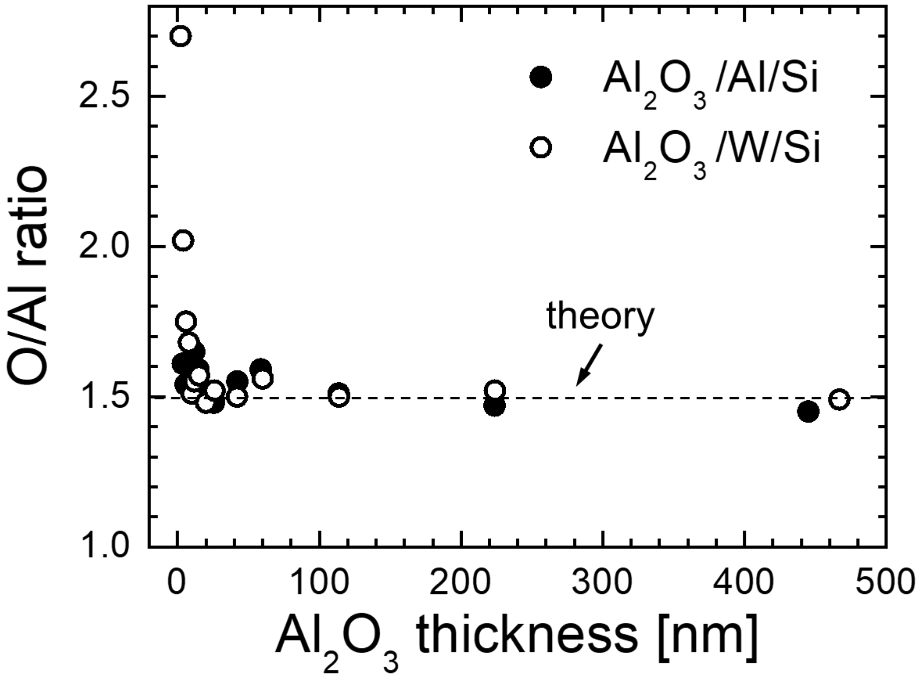

Figure S1 **Evidence for the alumina stoichiometry.** O/Al elemental for Al_2O_3/Al/Si and Al_2O_3/W/Si samples series plotted as a function of the Al_2O_3 thickness.

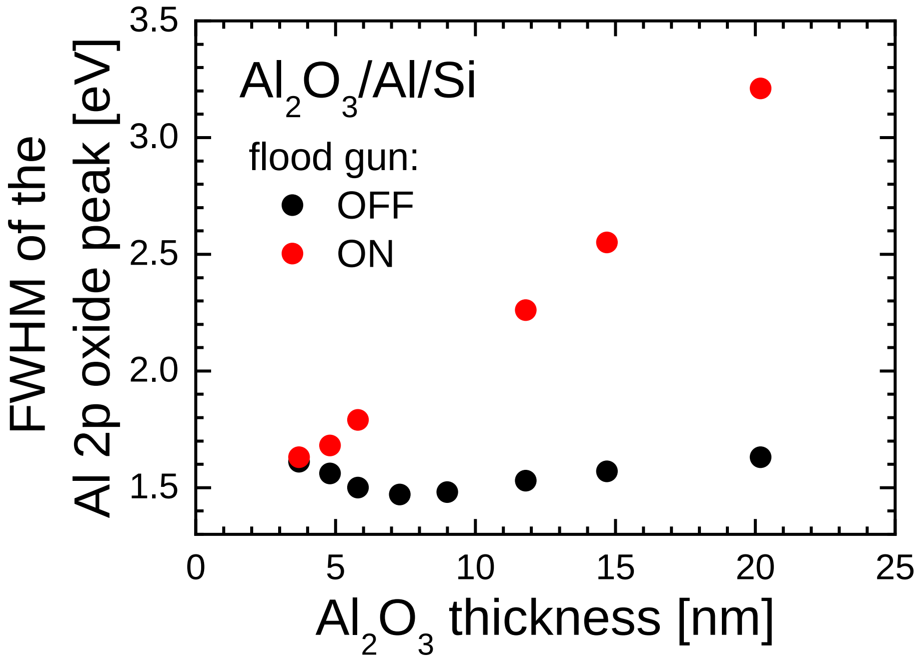

Figure S2 **Variation in the Al 2p peak width with increasing oxide thickness.** Full width at half maximum of the Al 2p oxide peak for Al_2O_3/Al/Si samples series plotted as a function of the Al_2O_3 thickness. Data points are showed for the measurements conducted with (red circles) and without (black circles) flood gun.

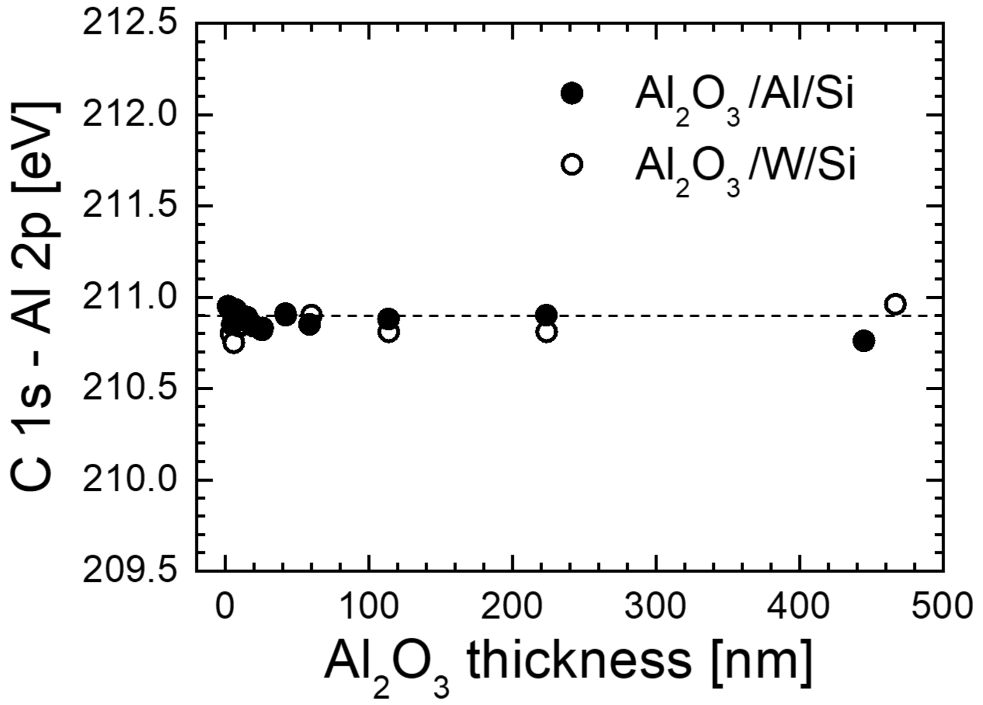

Figure S3 **Evidence for the constant binding energy difference between the C 1s peak of adventitious carbon and the Al 2p oxide peak from alumina films.** Binding energy difference between the C 1s peak from adventitious carbon (the C-C/C-H component) accumulating on top of Al_2O_3 and the Al 2p oxide peak from Al_2O_3/X/Si samples (X = Al or W). Results are shown for the alumina layer thickness in the range $2 \leq d_{Al_2O_3} \leq 467$ nm. Peak positions are measured without flood gun.

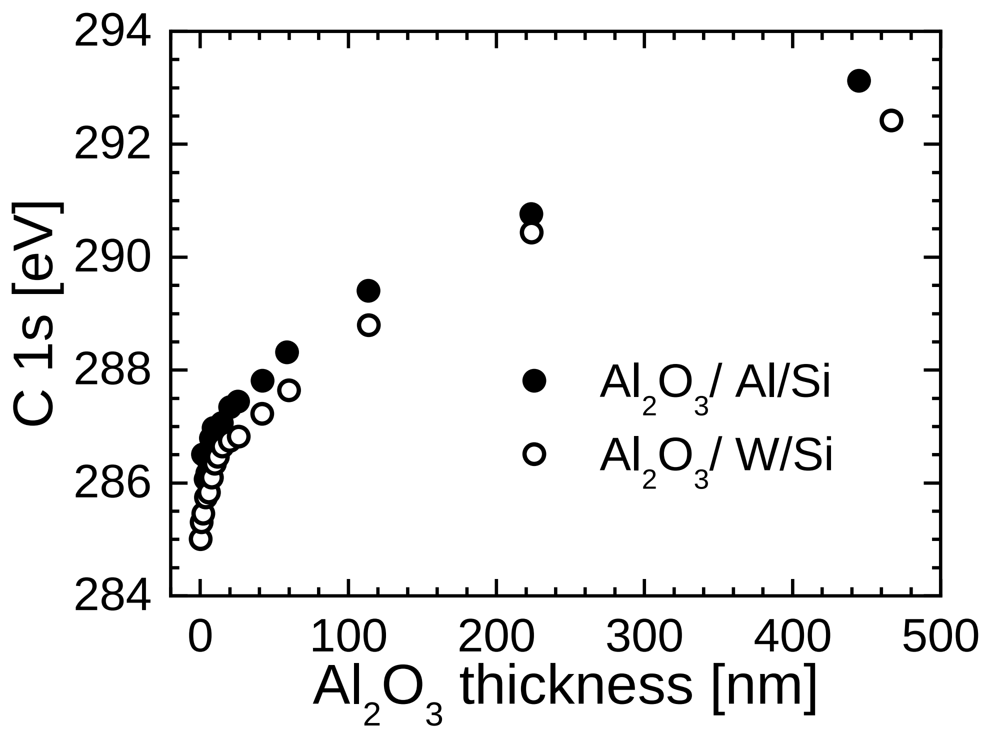

Figure S4 **Shifts of C 1s peak of adventitious carbon accumulating on top of alumina layers.** Binding energy of the C 1s peak from adventitious carbon (the C-C/C-H component) accumulating on top of Al_2O_3/X/Si samples (X = Al or W). Results are shown for the alumina layer thickness in the range $2 \leq d_{Al_2O_3} \leq 467$ nm. Peak positions are measured without flood gun.

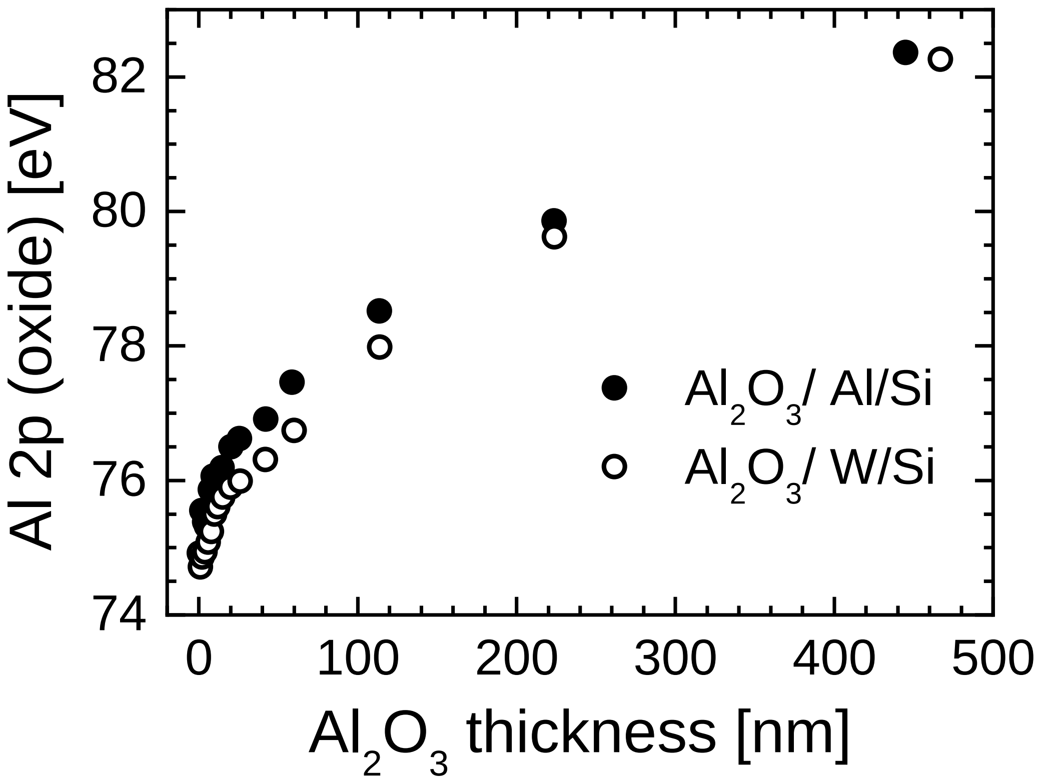

Figure S5 **Shifts of Al 2p peak of alumina.** Binding energy of the Al 2p oxide peak from Al_2O_3/X/Si samples (X = Al or W). Results are shown for the alumina layer thickness in the range $2 \le d_{Al_2O_3} \le 467$ nm. Peak positions are measured without flood gun.
